# Segmentation of infected region in CT images of COVID-19 patients based on QC-HC U-net

**DOI:** 10.1038/s41598-021-01502-0

**Published:** 2021-11-24

**Authors:** Qin Zhang, Xiaoqiang Ren, Benzheng Wei

**Affiliations:** 1 School of Computer Science and Technology, Qilu University of Technology, Jinan, 250301 China; 2 Center for Medical Artificial Intelligence, Shandong University of Traditional Chinese Medicine, Jinan, China

**Keywords:** Image processing, Machine learning

## Abstract

Since the outbreak of COVID-19 in 2019, the rapid spread of the epidemic has brought huge challenges to medical institutions. If the pathological region in the COVID-19 CT image can be automatically segmented, it will help doctors quickly determine the patient’s infection, thereby speeding up the diagnosis process. To be able to automatically segment the infected area, we proposed a new network structure and named QC-HC U-Net. First, we combine residual connection and dense connection to form a new connection method and apply it to the encoder and the decoder. Second, we choose to add Hypercolumns in the decoder section. Compared with the benchmark 3D U-Net, the improved network can effectively avoid vanishing gradient while extracting more features. To improve the situation of insufficient data, resampling and data enhancement methods are selected in this paper to expand the datasets. We used 63 cases of MSD lung tumor data for training and testing, continuously verified to ensure the training effect of this model, and then selected 20 cases of public COVID-19 data for training and testing. Experimental results showed that in the segmentation of COVID-19, the specificity and sensitivity were 85.3% and 83.6%, respectively, and in the segmentation of MSD lung tumors, the specificity and sensitivity were 81.45% and 80.93%, respectively, without any fitting.

## Introduction

Corona Virus Disease 2019 (COVID-19) is a pandemic and global disease. On February 11, 2020, the virus that causes the disease was named COVID-19 by the World Health Organization (WHO). As of May 10, 2021, the number of COVID-19 has exceeded 150 million, involving 223 countries or regions, including 3,288,455 deaths worldwide. To control the spread of the epidemic, Reverse Transcription Polymerase Chain Reaction (RT-PCR) is usually selected on the market to screen people infected with COVID-19^1^. However, RT-PCR is only used to determine whether the user is infected, and its accuracy is still inadequate. For example, in the recent outbreak in India, the RT-PCR was negative, but lung images showed signs of infection. Therefore, in addition to RT-PCR, other information needs to be integrated to make a judgment. In practice, lung Computer Tomography (CT) scan is a common and effective diagnosis and treatment methods. CT scan can show bilateral patchiness or ground-glass opacity (GGO) of the lung^2^, showing more pathological information with higher accuracy. In addition, it is convenient for doctors to make a detailed judgment based on the patient’s infection status, which is of positive significance to whether the patient is infected with COVID-19, and to establish the treatment plan. However, diagnosis and treatment depend on the judgment of doctors. In regions with severe epidemics and a lack of medical resources, the number of professional doctors is small, and the workload of the infected region is heavy when observed by naked eyes, which may lead to the effect of imaging diagnosis and treatment. To better deal with the problems caused by COVID-19, we need a tool to quickly and automatically segment the infected region of the lungs of COVID-19 patients^3–5^.It reduces the amount of work doctors have to do visually to separate infected areas, speeds up the diagnosis process and saves time on follow-up treatment.

At present, researchers are using artificial intelligence to achieve accurate image segmentation gradually mature. Due to Convolutional Neural Networks’s(CNN) strong feature extraction capability, more and more researchers choose CNN to analyze and process images^6^. Ordinary CNN fully connects the input layer and the hidden layer, but when it comes to a larger image, it is requires a lot of parameters to learn the features of the entire image through this fully connected method. Zhu et al. proposed a lightweight single-image super-resolution network EMASRN, and proposed a progressive multi-scale feature block to extract feature information of different sizes, and downsample the trained high-resolution(HR) image by using bicubic interpolation to synthesize low-resolution (LR) images^7^. The downsampling method based on interpolation improves the resolution of the image by the content of the image itself, without bringing more information, and has the side effects of noise amplification, increased computational complexity and fuzzy results. To improve this shortcoming, in the part of feature reconstruction network, Dense network is widely used, it provides an effective method of combining low-level features and high-level features^8^. Through the cascade of multiple Dense blocks, useful contextual information from a large area of the LR image is captured to restore high-frequency details in the HR image. However, Dense Block requires additional “transition” blocks to reduce feature mapping channels, which will result in additional parameters and a greater amount of calculation. Therefore, we learn the advantages of residual learning, and first combine the two to improve the network structure. And named it QC-HC U-Net. The innovations are as follows:

1. We combine the Dense network and Residual network and add them to the encoder and decoder respectively, forming a new quick connection. Compared with the Residual network and DenseNet, it has higher efficiency.
2. In the decoder of the network, Hypercolumns is added when up-sampling operation, which can accurately locate the information in the decoder and facilitate subsequent information extraction.
3. Since CT images of lung tumors have a high similarity with images of COVID-19 patients, MSD lung tumor cells were first used for training and testing in this network to verify the effectiveness of the model. Then, CT images of COVID-19 patients were selected for training and testing to obtain corresponding segmentation results. Practice proves that the improved result can get better segmentation results.

## Results

### Evaluation setting

*Quantitative evaluation*: According to commonly used evaluation indicators, this article uses complementary indicators to evaluate the results of this experiment, and the dice similarity coefficient is used to evaluate the overlap of regions. In addition, the widely used specificity and sensitivity is also used for evaluation^9^.

1 $$\begin{aligned} Dice(x,y)=\frac{2*|x\bigcap y|}{|x|+|y|} \end{aligned}$$

Among them, *y* is the image of the theoretical segmentation result, which is manually drawn by the expert, and *x* is the resulting image of the predicted segmentation.

2 $$\begin{aligned} Sensitivity=\frac{TP}{TP+FN} \end{aligned}$$

where *TP* is the number of positive classes predicted by the positive class, and *FN* is the number of negative classes predicted by the positive classes.

3 $$\begin{aligned} Specificity=\frac{TN}{TN+FP} \end{aligned}$$

Among them, *TN* is the number of negative classes predicted as negative classes, and *FP* is the number of negative classes predicted as positive classes.

*Qualitative evaluation:* We analyzed the segmentation results on the two datasets^10^. The segmentation of the tumor area is marked with color. In the COVID-19-CT-Seg datasets, the background area and the infected area are represented by colors. Under the same pixel, the result of our method makes the image produce a clearer outline of the infected area.

### Segmentation results

For improve the accuracy of the experiment, this paper compares the experimental results with the segmentation results of “Automated Chest CT Image Segmentation of COVID-19 Lung Infection based on 3D U-Net” proposed by Müller et al.^11^; and compared with the “Automated Chest CT Image Segmentation of COVID-19 Lung Infection based on 3D U-Net” proposed by Ma et al.^12^ Corresponding to the indicators in Muller’s paper, this paper adopts three broad indicators, namely dice similarity index (DSC), sensitivity (Sens), specificity (Spec).

The specific comparison results are shown in Tables 1 and 2. In terms of DSC and Senc, the segmentation results of the QC-HC U-Net adopted are superior to those of the other two 3D U-Nets. We compared the segmentation of infected regions in the Medical Segmentation Decathion (MSD) lung tumor data set, and the specificity was increased by about 8%. The segmentation of lung and infected areas in the COVID-19 patient CT image datasets (COVID-19-CT-Seg) was improved compared to other 3D U-Nets. The experimental results show that the addition of skip connections, shortcut connections and Hypercolumns among the structures provide reliable experimental proof for the optimization ability of the model. Table 1 shows the segmentation results of COVID-19-CT-Seg. Compared with the original 3D U-Net, the segmentation of DSC and Sens for tumor parts increased by about 6% and 3%, respectively. Since only the COVID-19-CT-Seg data set was used in the experiment of Müller et al.^13^, this article downloaded the code of Müller et al. and verified it with the MSD data set. To facilitate us to observe the changes of data, we chose to mark the best segmentation results in bold. The results are shown in Table 3.

According to the results in Tables 2 and 3, the segmentation results on COVID-19-CT-Seg are better than those of the other two 3D U-Nets. In the segmentation of the datasets, the segmentation of the lung backgroundand the segmentation of the infected region are all improved. Prove the effectiveness of the network architecture of this article.

**Table 1:** Comparison of segmentation indexes of COVID-19 pulmonary infection.

	DSC (%)	Sens	Spec
Ma’s Benchmark	67.3	–	–
Dominik’s 3D U-Net	79.92	81.15%	99.92%
QC-HC U-Net	**85.31**	**83.60**%	**99.96**%

**Table 2:** Comparison of pulmonary segmentation indexes of COVID-19.

	DSC (%)	Sens	Spec
Ma’s Benchmark	87.99	–	–
Dominik’s 3D U-Net	97.29	97.06%	**99.89%**
QC-HC U-Net	**98.64**	**98.14**%	99.88%

**Table 3:** Comparison of MSD tumor segmentation.

	DSC (%)	Sens	Spec
Ma’s Benchmark	67.72	–	–
Dominik’s 3D U-Net	73.20	77.66%	99.93%
QC-HC U-Net	**81.45**	**80.93**%	99.91%

Moreover, according to the data in the table, our segmentation results were superior to those of the other two methods, especially in the DSC and Sens, especially in the COVID-19-CT-Seg infection segmentation. This is mainly attributed to the optimization strategy adopted, which can achieve the extraction of feature information and effectively avoid the vanishing gradient, thus achieving good performance.

**Figure 1:**
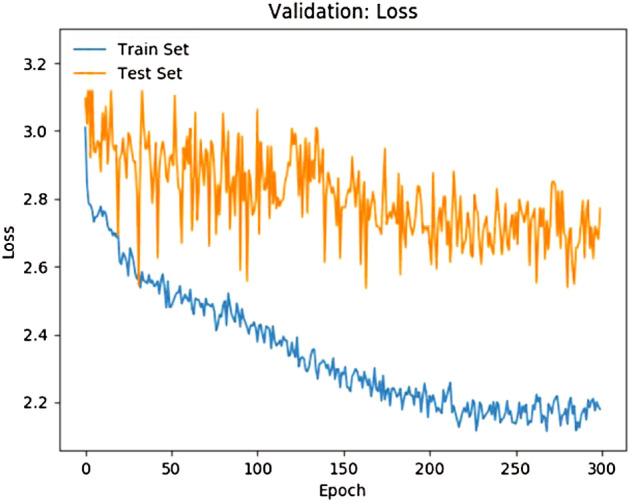
Loss function for training and testing.

According to the loss function we selected, it can be seen from Fig. 1 that in the process of iteration, the loss function tends to be stable with the increase of the number of iterations.

**Figure 2:**
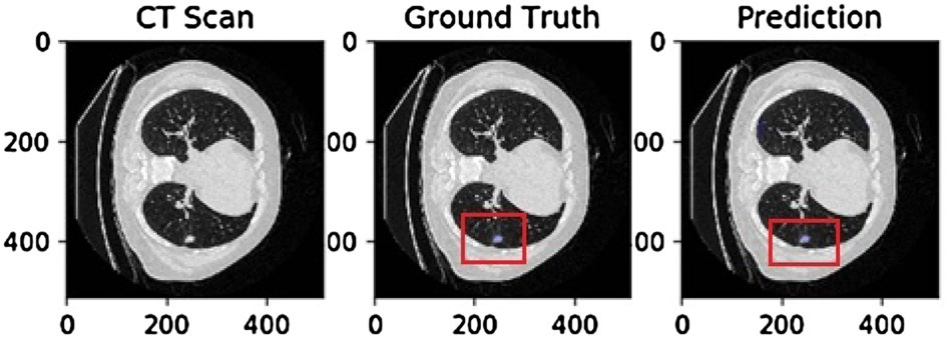
MSD lung tumor segmentation results.

**Figure 3:**
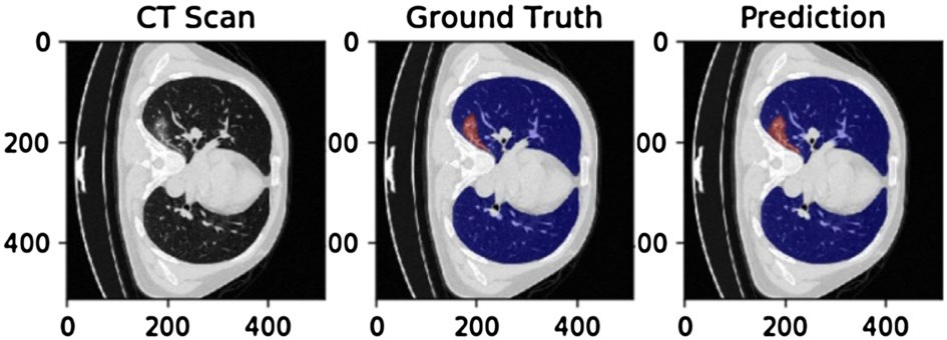
Segmentation results of COVID-19-CT-Seg patients when the number of slices is 139.

Image segmentation results are shown in Figs. 2 and 3, where the blue area is the experimental segmentation area. Since the tumor is too small to be seen clearly, it is circled in red in Fig. 2. The leftmost image is the original image, the middle image is the doctor-labeled image, and the rightmost image is the segmentation result of the experiment in this paper.

## Discussion

At present, there are many publicly available large data sets of COVID-19, as shown in Table 4, but most of these data are unannotated and cannot be trained in this experiment. Shan creatively proposed a “human-in-the-loop” (HITL) method, which adopts VB-NET network semantic segmentation^14^. This method can be trained according to a small part of the annotated data, generate new annotated data, and then train again. HITL can not only effectively alleviate the problem of insufficient data, but also solve the problem of image data requiring large-scale annotation. Another way to solve the data shortage is to transfer to learn from other data. Ahuja et al. proposed the COVID-19 is detected using transfer learning from CT scan images decomposed to three-level using stationary wavelet^15^. The above method is the self-annotation of data on the basis of relevant experiments^16–19^. Although the problem of insufficient data is solved to a certain extent, supervised learning has certain strict requirements for the judgment of accuracy, and relative comparison and analysis of labeled data and predicted data are required. Therefore, in order to ensure the effectiveness of the segmentation model, first of all, we still choose to train and test on the MSD data set, optimize the model, and then train and test again on the COVID-19-CT-Seg. Second, our experiment chooses to train in the MIScnn pipeline, which is mostly used for training on small data sets. There are two methods to alleviate the shortcomings of insufficient data.

**Table 4:** COVID-19 related dataset.

Dataset name	Type	Composition	Describes
COVID-19 CT segmentation dataset	CT	COVID-19	COVID-19 infected region
JSRT Dataset	X-ray	normal	Pulmonary region
COVID-19-Dataset	CT	COVID-19	349 CT images
COVID-19 BSTI Imaging Database	CT	COVID-19	COVID-9 imaging
COVIDx	X-ray	COVID-19 and no COVID-19	13975 X-ray images

Image segmentation is an important prerequisite for medical image analysis and interpretation. Due to the diversity and complexity of medical images, the segmentation performance of general models is often limited. Researchers have proposed a Deep Neural Network model suitable for medical image segmentation from multiple dimensions^20,21^. For example, He et al used the multi-task learning strategy to introduce the feature curve information into the feature learning, and applied it to guide the segmentation of the prostate, bladder and rectum^22^. In medical images, multimodal data can provide information at many levels due to different imaging mechanisms. Tang et al. designed a double-structure deep feature fusion Network for CT-multimodal MR image segmentation, and supplemented CT features with MR features at the level of advanced semantic features to improve the segmentation results of postoperative glioma on CT images^23,24^. For example, Zeng et al. proposed a deep supervised 3D U-Net Net-like full convolutional Network for the segmentation of proximal femur in 3D Magnetic Resonance (MR) images^25^. Compared with other networks, the U-Net network requires fewer parameters and is not easy to overfit. Therefore, U-Net and its variants (3D U-Net, U-Net ++, and Attention U-Net) are widely used in the field of medical image segmentation^25–28^.

U-net network is upsampled four times in total, and skip connection is used in the same stage, instead of directly monitoring and loss back transmission on advanced semantic features. In this way, more low-level features are integrated into the feature graph that is finally restored, and features of different scales are also integrated, so that multi-scale prediction and DeepSupervision can be carried out. Four upsampling also makes the edge recovery information of the segmented image more refined. Since the structure of the organ itself is fixed, semantic information is not particularly rich. So both high-level semantic information and low-level features are important (u-Net’s jump connections and U-shaped structures are more appropriate). The 3D U-Net we chose mainly lies in the fact that volumetric image does not need to input each section separately for training but can take the whole image as input to the model. These volumetric images are of great help, because it largely solves the embarrassing situation of 3D images being sliced into the model for training, and it also greatly improves training efficiency.

This article is dedicated to improving the segmentation accuracy and reducing the adverse effects of the segmentation results in the later stage. We combine the two methods of shortcut connection and Hypercolumns and apply them to the 3D U-Net network to improve the segmentation performance of the network.

According to the segmentation results, compared with 3D U-Net by Dominik et al. and Benchmark published by Ma et al. We added quick connection and Hypercolumns in the structure, and the DSC coefficient of “Comparison of segmentation indexes of COVID-19 pulmonary infection” increased by 18% compared with the Benchmark. This is an increase of about 6% compared to the 3D U-Net aspect of Dominik et al. The Senc index increased by 3.5% compared to the results of Dominik et al. All indicators on the MSD data set have also undergone corresponding growth. The specific results are shown in Tables 1, 2, and 3. In the “Comparison of segmentation indexes of COVID-19 Pulmonary infection” table, the Spec reached 99.96%, an increase of 0.04%. When segmenting the entire lung area, the Senc index dropped by 0.01%, and in the MSD data set, the Spec dropped by 0.02%. The experimental results prove that the misdiagnosis rate decreases with the increase of Spec, and the QC-HC U-Net network is more inclined to segment small areas. Sens is simply a better way of screening out people who are sick. Spec is screening out the normal ones. At the same time, the enhancement of intentionality and Sens (as shown in Table 1) means that the model has a better classification of the population (the negative and positive can be well distinguished). This proves that our model has certain advantages in the segmentation of COVID-19 lung infection regions. As shown in Tables 2 and 3 Spec indices, the model’s segmentation of the entire lung region and tumor region need to be improved. The reduced value makes the prediction result more likely to be positive, and TP and FP both increase. At the same time, the prediction result is Negative reduction, TN and FN synchronous reduction, which will lead to the reduction of Spec.

## Methods

Thisthesis is based on 3D U-Net, and we hope to achieve better performance by reducing model parameters. It can avoid the problem of gradient disappearance in the process of information transmission by adding skip connections. By establishing feed-forward connection^13^, the DenseNet can effectively transfer information at each layer to ensure the integrity of information transmission and can reduce the number of parameters on the basis of avoiding gradient disappearance. Combining the advantages of the two Network structures, this article establishes a new connection method—Quick connection, which is used in the 3D U-Net to reduce the parameters of the Network and also have better segmentation performance. And we choose to add Hypercolumns to accurately locate the information when performing up-sampling in the decoder, thereby reducing the loss of feature information.

### Datasets

Because the proportion of tumor cells and the lung infection in MSD images is relatively similar, and the information of lung proportion in CT images is relatively similar. We trained through MSD to verify and optimize the performance of the network to better train COVID-19 data.

#### Ethics declarations

We confirm that all experimental schemes have been approved by the licensing Committee of the school of computer science and technology of Qilu University of technology. We confirm that informed consent was obtained from all subjects or, if subjects are under 18, from a parent and/or legal guardian.

#### MSD lung tumor segmentation datasets

Using MSD lung tumor segmentation datasets, the datasets comes from Stanford University (California, USA). The dataset includes patients with non-small cell lung cancer and is a public data set. And this data set served as a segmentation challenge during MICCAI 2018. A total of 63 cases of CT scans, annotated to include lung cancer, were marked by a professional thoracic radiologist.

#### COVID-19-CT-Seg datasets

We used the data set selected by Ma et al.^12^. CT scans of 20 publicly available COVID-19 patients, containing more than 1, 800 annotated sections, were collected from the Coronacases Initiative and Radiopaedia, which contained the CC By-NC-SA license. The annotations were annotated by primary annotations, refined by two doctors with 5 years of experience, and finally, verified by one doctor with 10 years of experience. Notes include left lung, right lung, background, and infection components, but the primary markers of infection are selected.

### Data processing

We select the following three preprocessing methods: data normalization, data standardization and resampling. And the method of data enhancement is used to improve the situation of insufficient data so that the segmentation results of the model can be more accurate.

#### Normalization and standardization

Since COVID-19 data sets come from different sources, and the Hounsfield units(HU) of the infected region are $+50$ to $+100$, while the HU value of the lungs is $-1000$ to $-700$, we chose HU as the unit index to cut the pixel intensity value of the image to a maximum of $+250$ and a minimum of $-1250$. Because the pixel values of CT images derived from Radiopaedia are already within the normal range of 0 to 255. We just adjusted the CTs image from the Coronacases Initiative so that its pixel value was also within the corresponding range. In image processing, the range of pixel values of the transformed image can affect the fitting process and segmentation results, so, we chose Z-score, a common standardized method, for all the imaging sample data.

#### Resampling

A common parameter on medical images is voxel spacing, which translates the distance among two adjacent points in the image into a representation of the distance between the volumes. However, the voxel spacing in the lung images is not fixed. To reduce the complexity in the training process and capture the context information effectively, the image datasets were resampling, and the voxel spacing was $1.58 \times 1.58 \times 2.70$, and the volume shape was $267 \times 254 \times 104$.

#### Data enhancement

To make up for the lack of data, we chose the following four types of data enhancement to augment the datasets. Furthermore, each augmentation method had a random probability of 15% to be applied on the current image with random intensity or parameters (e.g.random angle for rotation). Through this technique, the probability that the model encounters the exact same image twice during the training process decreases significantly.

1. Space expansion
  We mainly choose to expand the space by mirroring, rotating, scaling and elastically deforming the image. When the image is changed, the size of the image will change. At this time, choose the simplest difference value and fill the unknown region with some constant values.
2. Grace
  We mainly increase the color by changing the gamma value, brightness and contrast. The sampling range of gamma value is selected (0.5, 2), the range of brightness is sampled from the multiplier range (0.5, 2), and the same brightness the modifier is used for all colors.
3. Noise
  We choose to add Gaussian noise to enhance the image data. The noise variance is randomly selected from (0, 0.1).
4. Patchwise
  This method can reduce the risk of overfitting. To facilitate our research on the data, we randomly crop the image and cut the image roll into $160 \times 160 \times 80$ patches, which is convenient for us to study the data. Among these patches, we introduced half of the size of the patch ($80 \times 80 \times 40$) to overlap them to improve the performance of prediction. After each patch is predicted, they are reconstituted into the volume of the original shape, and the overlapping region will be divided equally at this time. Through the above data processing methods, the problem of insufficient data can be greatly improved, and the model can be better optimized.

### Network structure

#### Network overview

**Figure 4:**
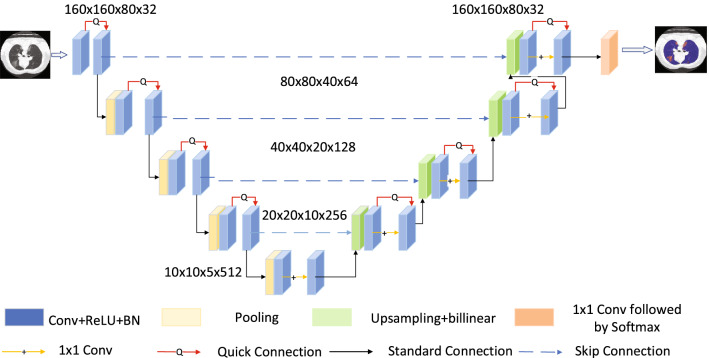
3D U-Net architecture diagram.

The 3D U-Net used is shown in Fig. 4. The Network is mainly divided into encoder and decoder. The encoder consists of convolution, Batch Normalization (BN), Rectified Linear Unit (ReLU) and Polling, which can extract features from the input images and analyze them. Decoder is composed of up-sampling, convolution, BN (Batch Normalization) and ReLU and its function is to generate a segmenting block map after receiving the analyzed image of the encoder. The highest resolution of the network uses 32 feature maps, and the lowest resolution uses 512 feature maps. In order to achieve better applicability of the Network and effectively avoid the disappearance of the gradient, this article chooses to add skip connections in the encoder and decoder, and the encoder information can be directly transmitted to the decoder; To reduce the loss of information in the combination of characteristic information, we add a quick connection to each convolution block, which is represented by the red line in Fig. 4. In order to accurately locate the information in the decoder and enable better extraction of information, this article chooses to add Hypercolumns when up-sampling is performed in the decoder.

#### Quick connection

**Figure 5:**
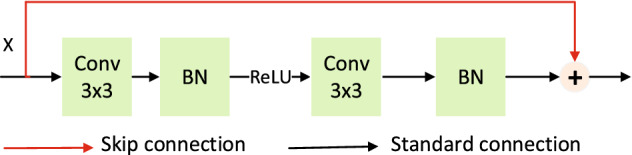
ResNet module.

ResNet is a widely used network architecture. It differs from 3D U-Net in that ResNet adds a connection between Convolutional and Rectified Linear Unit(Conv-ReLU) and Conv-ReLU, as shown in Fig. 5. A skip connection is added to this network, which is equivalent to taking a shortcut when carrying out output transmission and skipping the operation of the current layer. In the process of back-propagation, the network information of the next layer is also skipped operation and directly transferred to the next layer (all 1:1 transmissions, no additional parameters are required). The advantage of this approach is that it effectively avoids the gradient disappearance caused by the operation.

**Figure 6:**
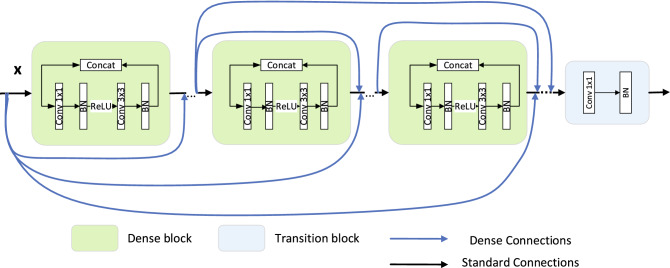
Dense block.

However, the skip connection has an obvious shortcoming in that it lacks the dense concatenation of the convolution output feature map. It means that when the input information passes through many layers, the information will be lost before reaching the end, which is not conducive to the accuracy of the result. To avoid the loss of information and ensure the maximization of information dissemination, the DenseNet proposes a more radical dense connection mechanism: all the layers are interconnected. Specifically, the input of each layer of the network is the union of the output of all the previous layers, and the feature graph learned by this layer will be directly transmitted to all the layers behind as input. In this way, each layer contains the output of all previous layers. Since DenseNet directly contacts across channels, the size of feature graphs for different layers should be the same as contact. To facilitate contact, DenseNet divides multiple Dense blocks. Because the size of the feature map inside each Dense block is not the same, it chooses to use a 1x1 convolution operation to aggregate the feature maps, and crop the input information to fit the layer output. This is shown in Fig. 6. The feature map matching of the exterior of the Dense block chooses to use a Transition module between each Dense block to make the excessive connection. These features allow DenseNet to achieve better performance than ResNet with less parameter and computing costs. DenseNet can alleviate the problem of gradient disappearance, and it can also greatly reduce the number of parameters and enhance the propagation of features. DenseNet dense connection is shown in Fig. 6:

However, Densenet will carry out many concatnate operations, which requires a large amount of storage and some storage optimization techniques. Especially when the number of DenseNet layers is larger, the storage required is even greater.

Inspired by Jafari et al., we also added shortcut connections in 3D U-Net^13^. First of all, a shortcut connection is added between the first Conv-BN(Convolutional and Batch Normalization) and the last Conv-BN output, and then the result of the connection is summed up with the operation result through the Conv-BN to achieve the function of aggregation feature graph like the DenseNet. This connection allows the parameters to be updated at the first Conv and if the gradient of the subsequent Conv is close to zero, allows subsequent operations to proceed without the gradient loss. Secondly, unlike the DenseNet that selects multiple learnable 1x1 Convs to aggregate the feature maps, this article chooses to use a simple summation method to combine the feature maps.

**Figure 7:**
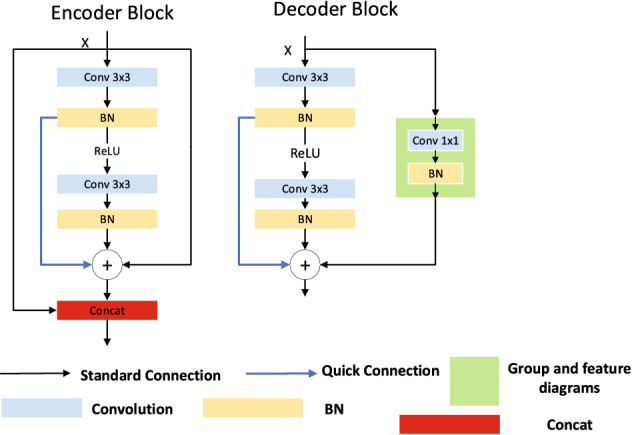
Quick connection.

In this article, input and output in each layer of the encoder section are combined in a cascading manner, and then the result of the feature graph combination is transmitted to the next layer (Fig. 7 encoder Block). This has the advantage of having input information from each layer and allowing the feature graph to match the feature graph of the next layer with fewer parameters. In the cascade block added in this article in the decoder part, the Conv $1 \times 1$ and BN algorithms are used to perform operations (as shown in Fig. 7 decoder Block). Compared with the contact operation in the DenseNet, this method can reduce the number of input channels.

#### Hypercolumns

On the basic 3D U-Net architecture, Hariharan et al. Proposed^29^ that the features of the last layer of the decoder is relatively rough, while the information of the first layer of the decoder is too precise and lacks semantic information. Therefore, they choose to add Hypercolumns when performing up-sampling operations on the decoder, and to weigh the information between the different layers of the decoder part. The specific method is to compare all nodes of the corresponding pixel Network Activate the output value as a feature in order to locate the target. The principle is to form a vector of the position activation output values of all corresponding pixels for later operation. This paper uses the above methods for reference to improve the segmentation accuracy.

We extract the output on the corresponding layer by simply adjusting each feature map to the size we want by using bilinear interpolation. The sampled feature map is used to represent *F*, then the feature vector of the *i* position is as required by Eq. (4):

4 $$\begin{aligned} f_{i}=\sum _{k} \partial _{ik} F_{k} \end{aligned}$$

where *f* is the feature graph of upsampling, and $f_i$ is the feature vector of this position. $\alpha$ $_{ik}$ depends on the position of *i* and *k* in the box and feature map.

Since these feature mappings are the result of convolution, they do not encode the boundary information of a given image pixel. We can take advantage of the nonlinear effect of this position, but we cannot infer it from the deviation of a particular position. This requires a different classifier for each location. The easiest way to get a location-specific classifier is to train each individual classifier for 5050. Train the kk grids of the classifier and insert functions $g_k$($\cdot$) between them. Each classifier is a function $g_k$ that takes an eigenvector and outputs the probability between 0 and 1. As shown in the specific Eq. (5):

5 $$\begin{aligned} h_{i}(\cdot )=\sum _{k} \alpha ^{(j)}_{ik} F^{(j)}_{k} \end{aligned}$$

where $p_{ik}$ is the probability output of the *K* classifier at the position. The above process is to adjust the size of all feature maps and then classify each position. However, it would be too much to calculate hundreds of feature maps based on this method, so we can split the super columns at relevant positions and then perform the calculation.

6 $$\begin{aligned} p_{i}(\cdot )=\sum _{k} \alpha _{ik} g_{k}(f_{i})=\sum _{k} \alpha _{ik} p_{ik} \end{aligned}$$

We will run several linear classifiers at the top of the high-level functionality, and we will save the feature mapping calculation by using the following operations. As shown in Eq. (7), the corresponding weight block is set in the classifier.

7 $$\begin{aligned} W^{T}f_{i}=\sum _{j} W^{(j)T} f^{(j)}_{i} \end{aligned}$$

**Figure 8:**
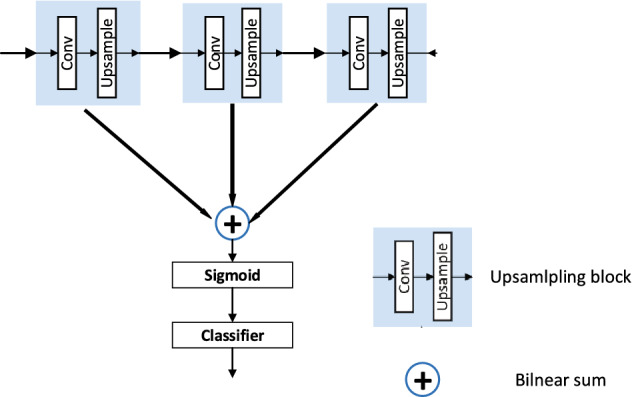
Hypercolumns schematic.

$f_i$ is the feature vector at position *i* and consists of the corresponding *j*th feature map $F^{(j)}$. *w* is a linear classifier, and it is decomposed. The classifier *w* is $w^{(j)}$ after upsampling by Eq. (4), and $f_i^{(j)}$ is expressed as *f* (*j*) after upsampling by Eq. (4). Since the up-sampling is a linear operation, we can apply the classifier first. Observe that applying the classifier to every position in the feature map is the same as a 11 convolution. Therefore, we decompose it into blocks corresponding to each feature map and run a 11 convolution on each feature map to generate a fractional graph so that all fractions map to the sum of the target resolution. As shown in Eq. (8). The schematic diagram is shown in Fig. 8.

8 $$\begin{aligned} W^{(j)T}f^{(j)T}=\sum _{k} \alpha ^{(j)}_{ik} F^{(j)}_{k} \end{aligned}$$

#### Experimental design

The experiment was chosen to be carried out on the Tensorflow framework. Since the infection of COVID-19 is similar to lung tumors, this article first selects 80% of the MSD lung tumor dataset for training and 20% for testing, and continuously optimizes the model. Finally, 80% of the COVID-19-CT-Seg data is also selected for training and 20% for testing, so that the lung infection region of COVID-19 can be segmented more accurately. Finally, 80% of the COVID-19-CT-Seg data is also selected for training and 20% for testing, so that the lung infection region of COVID-19 can be segmented more accurately. We performed 150 iterations for each dataset, with a batch size of 2 and an epoch of 300, and the whole process for each dataset took about 30 h. This article uses the Adam optimizer for training, and the learning rate is set to 0.0001. In order to avoid training loss, this article also adopts a dynamic learning rate, which can reduce the learning rate by 0.1.

Since the proportion of the infected region is small, it is not conducive to accurate segmentation. Therefore, this paper uses the sum of the Tversky index^30^ and the classification cross-entropy as the loss function Eqs. (9, 10, 11).

9 $$\begin{aligned} L_{total}= & {} L_{CCE}+L_{Tversky} \end{aligned}$$

10 $$\begin{aligned} L_{CCE}= & {} -\sum _{c=1}^N y_{o,c}log(P_{o,c}) \end{aligned}$$

11 $$\begin{aligned} L_{Tversky}= & {} N-\sum _{c=1}^N \frac{TP_{c}}{TP_{c}+\alpha \cdot FN_{c}+\beta \cdot F} \end{aligned}$$

*FP* means false positive, and *FN* means false negative. False negatives and false positives can be controlled, and the trade-off between false positives and false negatives can be carried out through control. In this experiment, 0.5 is selected. The cross-entropy Loss function belongs to the Loss function of classification type, and the element only has two values of 0, 1, which can be used in the scene of semantic segmentation on a large scale. The multi-class adaptation for multiple categories (categorical cross-entropy) is represented through the sum of the binary cross-entropy for each class *c*,whereas $y_{o,c}$ is the binary indicator of whether the class label *c* is the correct classification for observation *o*.The variable $p_{o,c}$ is the predicted probability that observation *o* is of class *c*.

### Limitations

There are few COVID-19 data available for deep learning. To achieve the better training effect, this paper uses the method of data enhancement to increase the data set and verifies the effectiveness of the algorithm through MSD data before conducting COVID-19 analysis. Through the above methods, the model in this paper has achieved a relatively ideal result. Unfortunately, datasets are scarce. So having a lot of real data can further optimize the test model.

## Conclusion

In this paper, a QC-HC U-Net is designed. In terms of training data set, we firstly expand COVID-19 data by means of data enhancement to improve the problem of insufficient COVID-19 data. Then the data is preprocessed by normalization and standardization. In the aspect of model design, based on 3D U-Net, quick links are added in the modules of encoder and decoder to ensure feature extraction and effectively avoid the disappearance of the gradient. In the up-sampling operation of the decoder, simple Hypercolumns is added to facilitate the precise positioning of the decoder and the balance of the characteristic information between each layer of the decoder. We chose to test the model using datasets of lung tumor cells and COVID-19-CT-Seg, respectively. Compared with the benchmark of Ma et al. and the results of Muller et al., the model we designed outperforms the original network structure and provides better segmentation results.

## Data Availability

We hereby specifically state that all methods are carried out in accordance with the relevant guidelines and regulations. The COVID-19-CT-Seg we used contains an Attribution-NonCommercial-ShareAlike International (CC BY-NC-SA) certificate. The data set is made by Coronacases Initiative and Radiopaedia disclosure of open data sets, which is available at https://zenodo.org/record/3757476#. The MSD Lung Tumor data is provided by Medical Segmentation Decathlon, and the following descriptions are made about the data:All data will be made available online with a permissive copyright-license (CC-BY-SA 4.0), allowing for data to be shared, distributed and improved upon. All data has been labeled and verified by an expert human rater and with the best effort to mimic the accuracy required for clinical use.
